# Combinatorial Nanomedicine Made of Squalenoyl-Gemcitabine and Edelfosine for the Treatment of Osteosarcoma

**DOI:** 10.3390/cancers12071895

**Published:** 2020-07-14

**Authors:** Carlos Rodríguez-Nogales, Haritz Moreno, Carolina Zandueta, Didier Desmaële, Fernando Lecanda, Patrick Couvreur, María J. Blanco-Prieto

**Affiliations:** 1 Chemistry and Pharmaceutical Technology Department, School of Pharmacy and Nutrition, Universidad de Navarra, 31008 Pamplona, Spain; crodriguez.31@alumni.unav.es; 2 IdiSNA, Navarra Institute for Health Research, 31008 Pamplona, Spain; hmmoreno@unav.es (H.M.); czandueta@unav.es (C.Z.); flecanda@unav.es (F.L.); 3 Solid Tumors Program, Division of Oncology, Centre for Applied Biomedical Research (CIMA), University of Navarra, 31008 Pamplona, Spain; 4 Institut Galien Paris Sud, CNRS UMR 8612, Université Paris-Saclay, 92296 Châtenay-Malabry, France; didier.desmaele@u-psud.fr; 5 Department of Pathology, Anatomy and Physiology, School of Medicine, University of Navarra, 31008 Pamplona, Spain; 6 Centro de Investigación Biomédica en Red de Cáncer (CIBERONC), 28029 Madrid, Spain

**Keywords:** osteosarcoma, chemotherapy, pediatric cancer, metastasis, nanomedicine, gemcitabine, edelfosine, squalenoyl–gemcitabine

## Abstract

Due to chemoresistance and a high propensity to form lung metastasis, survival rates in pediatric osteosarcoma (OS) are poor. With the aim to improve anticancer activity in pediatric OS, a multidrug nanomedicine was designed using the alkyl-lysophospholipid edelfosine (EF) co-assembled with squalenoyl–gemcitabine (SQ–Gem) to form nanoassemblies (NAs) of 50 nm. SQ–Gem/EF NAs modified the total Gem pool exposure in the blood stream in comparison with SQ–Gem NAs, which correlated with a better tolerability and a lower toxicity profile after multiple intravenous administrations in mice. For in vivo preclinical assessment in an orthotopic OS tumor model, P1.15 OS cells were intratibially injected in athymic nude mice. SQ–Gem/EF NAs considerably decreased the primary tumor growth kinetics and reduced the number of lung metastases. Our findings support the candidature of this anticancer nanomedicine as a potential pediatric OS therapy.

## 1. Introduction

Pediatric osteosarcoma (OS) is a primary osseous malignancy, predominantly affecting patients younger than 20 years old [1]. It usually originates from primitive bone-forming mesenchyme of the knee region, between the distal femoral and the proximal tibial metaphyseal area [2]. Children and adolescents diagnosed with OS in 2020 are still treated with chemotherapeutic drug regimens that have remained mostly unmodified since the consolidation of methotrexate, doxorubicin and cisplatin treatment in the early 1980s [3]. A complete tumor resection together with adjuvant chemotherapy allows for a successful eradication of localized OS; however, lung metastasis represents the most common fatal outcome, with dismal prognosis [4]. Even though current protocols result in an overall 5-year survival of 70–80%, these rates decrease to 20% in patients with metastasis [5]. In addition, pediatric OS is characterized by a high complexity in genomic alterations [6,7]. Cancer cells often present a high predisposition to germline mutations in the RB1 and TP53 tumor-suppressor genes, as well as, oncogene overexpression of MET, MYC and FOS, among others [8,9].

Having in mind the limited role of radiation and immunotherapy in the treatment of OS to date, the administration of high doses of triple-drug chemotherapy combinations represents the last chance to prevent tumor recurrence [10]. Unfortunately, these regimens frequently meet with poor tolerance, resistance and long-term toxicities with pernicious morbidity in children [11,12].

Cancer nanomedicine has emerged to improve the therapeutic index of chemotherapeutic drugs [13,14]. Gemcitabine (Gem) (Gemzar^®^) is a second-line antineoplastic agent given in pediatric OS which has been amenable in nanomedicine [15]. Although it is extremely active against a wide variety of tumors, this nucleoside analog (2’,2’-difluorodeoxycytidine) is rapidly inactivated by cytidine deaminase [16]. Squalenoyl–gemcitabine nanoassemblies (SQ–Gem NAs) have been reported to dramatically increase the low half-life of Gem in vivo, yielding improved efficacy in several tumor models [17,18]. However, in some cases this longer cytotoxic drug exposure entails a myelosuppression that can restrain the tolerability for SQ–Gem NAs than free Gem [19]. We anticipate that this toxicity may be exacerbated especially in the pediatric population, being detrimental in high-risk patients [20].

To circumvent this limitation, we performed the insertion of the alkyl-lysophospholipid edelfosine (EF) within SQ–Gem NAs. EF is a pro-apoptotic drug that does not damage the DNA integrity, in contrast to Gem. Its antitumor activity relies on the activation of the Fas/CD95 cell death receptor via lipid rafts or inhibition of the MAPK/ERK and the Akt/protein kinase B survival pathways [21,22]. We previously reported that the amphiphilic structure of SQ–Gem and EF allowed for their complete equimolecular co-assembly by an easy nanoformulation process, resulting in the formation of stable multilamellar NAs of 50 nm with unique physicochemical features (e.g., distinct spatial arrangement) in comparison with SQ–Gem NAs [23]. By means of this new molecule interaction, EF is thought to enhance SQ–Gem tolerability by modifying its pharmacokinetic behavior in the blood stream. This approach may be optimal for increasing the doses in pediatric solid tumors such as OS, diminishing potential toxic effects.

**Scheme 1:**
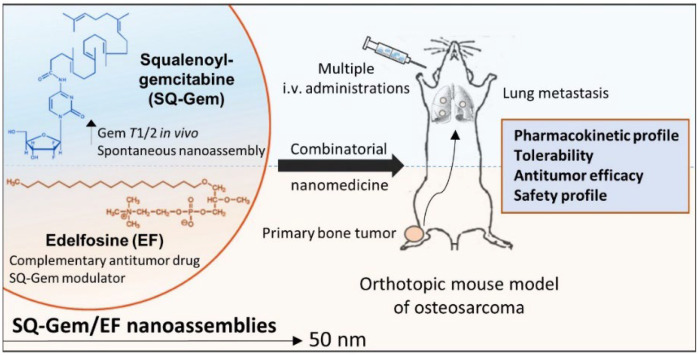
Schematic diagram of the squalenoyl–gemcitabine (SQ–Gem)/edelfosine (EF) nanoassemblies (NAs) for the pediatric osteosarcoma (OS) treatment.

All-in-all, the aim of the current study was to assess the therapeutic impact resulting from the incorporation of EF into SQ–Gem NAs for the treatment of high-grade pediatric OS (Scheme 1). Using an in vivo OS model, we discovered that multiple administrations of SQ–Gem/EF NAs were better tolerated than SQ–Gem NAs and delayed the progression of the primary tumor as well as its metastatic lung dissemination.

## 2. Results

### 2.1. Preparation and Characterization of SQ–Gem and SQ–Gem/EF NAs

Prior to the in vitro and in vivo studies, SQ–Gem NAs and multidrug SQ–Gem/EF NAs were prepared by nanoprecipitation. As shown in Table 1, the mean particle diameter of SQ–Gem NAs was measured to be around 116 nm with a unimodal size distribution. Since each Gem molecule was chemically linked to the squalenic acid, the total drug content was 40.7% according to the molecular weights. The particle population of the SQ–Gem/EF NAs was also very homogeneous, but with a smaller mean particle size diameter of only 50 nm. The full equimolecular co-assembly of EF into SQ–Gem NAs was previously confirmed by UHPLC/MS/MS [23], leading to a total drug content of 67.28% (44.78% for EF and 22.49% for Gem). We must bear in mind that once the equimolar ratio was surpassed, it was not possible to decrease SQ–Gem/EF NAs mean particle diameter further and assert a complete co-encapsulation of EF within SQ–Gem.

### 2.2. SQ–Gem and SQ–Gem/EF NAs Displayed a Similar Anticancer Activity In Vitro

**Figure 1:**
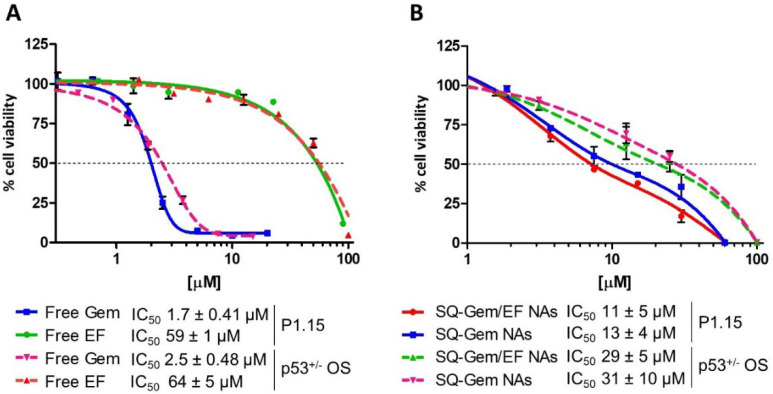
In vitro cytotoxicity representative curves of (**A**) free Gem versus free EF and (**B**) SQ–Gem NAs versus SQ–Gem/EF NAs treatments tested on p53^+/−^ OS and P1.15 cells. IC_50_ values are the mean ± SD of at least three independent determinations.

Two OS cell lines, c-Fos overexpressing P1.15 and primary OS cells (p53^+/−^ OSs), were initially screened to compare the in vitro antiproliferative activity of the free drugs (i.e., Gem and EF) versus their nanoparticle counterparts. In both cell lines, IC_50_ values corresponding to the free drug were very similar, although Gem (1.7 and 2.5 µM, respectively) was shown to be much more cytotoxic than EF (59 and 64 µM, respectively) (Figure 1A). As observed in Figure 1B, SQ–Gem NAs IC_50_ values were 6 and 12 times higher than those resulting from Gem treatment, which was expected due to the “pro-drug” effect of SQ–Gem, the release of Gem from the NAs being mandatory to exert any cytotoxic activity (13 and 31 µM in P1.15 and p53^+/−^ OSs, respectively). SQ–Gem/EF NAs at equimolar concentrations barely decreased the IC_50_ values of SQ–Gem NAs given the negligible antiproliferative effects of EF in that concentration range. Nevertheless, SQ–Gem/EF NAs exhibited a better anticancer profile in P1.15 cells than in p53^+/−^ OSs cells (Figure 1B, continued lines vs. dotted lines), displaying IC_50_ values of 11 and 29 µM, respectively.

### 2.3. SQ–Gem/EF NAs Drastically Diminished the Total Gem Pool Exposure in the Blood Stream in Comparison with SQ–Gem NAs

**Figure 2:**
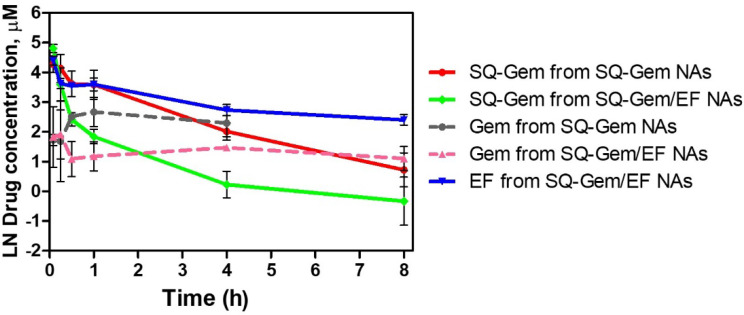
Plasma concentrations of Gem, SQ–Gem and EF after a single administration via retro-orbital plexus of SQ–Gem NAs or SQ–Gem/EF NAs (58 μmol/kg (Eq. to EF and SQ–Gem), as a function of time post-injection. Values are means ± SD (*n* = 3).

SQ–Gem/EF NAs and SQ–Gem NAs were injected via retro-orbital plexus in healthy mice to compare to their respective pharmacokinetic profile. Plasmatic concentration-time curves depicted in Figure 2 showed that the co-assembly of EF (blue curve) with SQ–Gem (green curve) significantly modified the pharmacokinetic behavior of Gem, as compared with SQ–Gem NAs (red curve). Indeed, the total amount of Gem in the blood stream (SQ–Gem + Gem released from SQ–Gem) was decreased after administration of SQ–Gem/EF NAs comparatively to SQ–Gem. The AUC_0-inf_ value for SQ–Gem from the SQ–Gem NAs treated group was 2.25-fold higher in comparison with SQ–Gem from SQ–Gem/EF NAs (88 versus 39 μg·h/mL), and correlated inversely with a total systemic clearance of 462 instead of 948 mL/kg/h (Table 2). In addition, Gem released from SQ–Gem NAs displayed a higher cleavage than that of Gem released from SQ–Gem/EF NAs during the first 4 h (gray dotted line versus pink dotted line). However, their AUC_0-inf_ values were similar (21 and 17 μg·h/mL, respectively) because the presence of Gem released from SQ–Gem/EF NAs in the blood stream was prolonged up to 8 h. Gem from SQ–Gem concentrations in plasma after 8 h are not depicted because values were below the limit of quantification of the technique (<1 μg/mL). Other relevant pharmacokinetic parameters are listed in Table 2, including those for EF from SQ–Gem/EF NAs.

### 2.4. The Insertion of EF into SQ–Gem Increased NAs Tolerability

**Figure 3:**
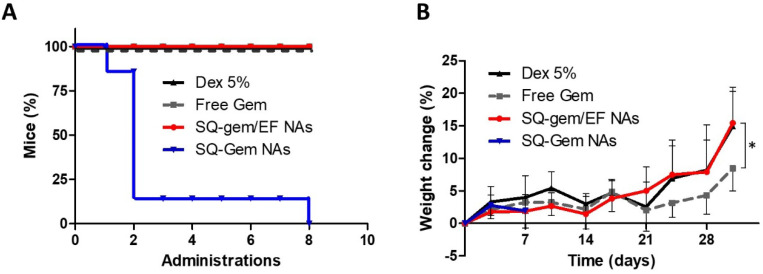
(**A**) Survival curves after 8 administrations of Dex 5%, free gemcitabine (Gem), squalenoyl–gemcitabine/edelfosine nanoassemblies (SQ–Gem/EF NAs) or SQ–Gem NAs at 38 μmol/kg (Eq. to EF, SQ–Gem and Gem) in tumor-bearing mice; (**B**) body-weight change over time (days). Values are means ± SD (*n* = 7), * *p* < 0.05.

To assess the influence of inserting EF into SQ–Gem NAs, we then investigated the tolerability of the SQ–Gem/EF NAs versus SQ–Gem NAs after multiple administrations in tumor-bearing mice. The doses used in the pharmacokinetic study were reduced from 58 to 38 μmol/kg in order to observe any subacute toxic effects associated with the long-term treatment. SQ–Gem NAs triggered a marked toxicity after the second administration, being lethal for 85% of the mice (Figure 3A). Interestingly, no fatal adverse events were found in the SQ–Gem/EF NAs treatment group during the experiment. Similarly, free Gem and non-treated (Dex 5%) groups did not display any mortality. Of note, no loss in body weight was detected in any of the groups (Figure 3B), confirming that SQ–Gem/EF NAs were well tolerated at least after eight consecutive administrations. Only the mean weight gain of mice receiving free Gem was slowed down from day 21. At day 32, this weight gain difference reached 7%, being statistically significant in comparison with the non-treated and SQ–Gem/EF NAs groups (* *p* < 0.05).

### 2.5. SQ–Gem/EF NAs Delayed the Progression of the Primary Tumor Growth and Lung Metastases in a P1.15 Orthotopic OS Model

**Figure 4:**
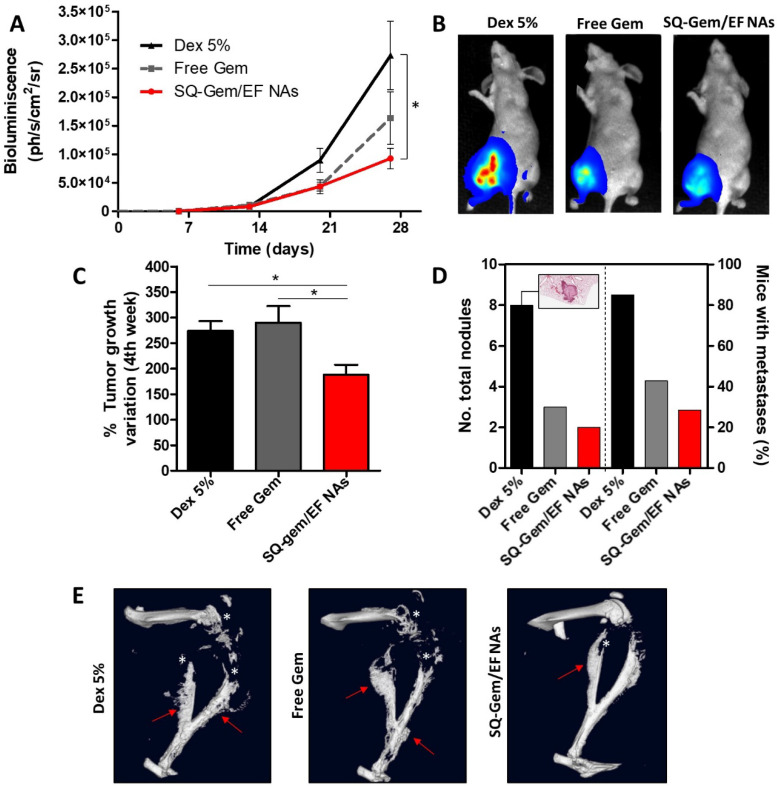
Tumor growth inhibition after multidrug treatment of mice bearing OS cells. All groups received eight intravenous injections in the retro-orbital plexus of either SQ–Gem/EF NAs, free Gem or a Dex 5% solution at the dose of 38 μmol/kg (Eq. to EF, SQ–Gem and Gem). (**A**) Hindlimb bioluminescence was regularly measured during the experimental period. The values are the mean ± SEM (*n* = 7) normalized to day 0; (**B**) representative bioluminescence images of OS bearing mice 27 days after the intratibial inoculation of P1.15 cells; (**C**) percentage of tumor growth variation measured as hindlimb bioluminescence signal corresponding to the 4th week (days 20 to 27) (*n* = 7, data = mean ± SEM, * *p* < 0.05); (**D**) quantification of the number of total metastatic nodules and the percentage of mice presenting macroscopic lung metastases (*n* = 7). A representative photomicrograph of a hematoxylin and eosin-stained histological section of lungs of a non-treated tumor-bearing mouse is shown; (**E**) representative micro-CT 3D images of the tumor-bearing hindlimbs for each treatment group. Red arrows: ectopic bone formation, white asterisks: osteolytic regions.

The anticancer activity of SQ–Gem/EF NAs was evaluated in a P1.15 orthotopic OS tumor model. Tumor growth kinetics in hindlimbs was monitored by bioluminescence imaging (BLI), during the course of the study, for SQ–Gem/EF NAs, free Gem and Dex 5% (control group) (Figure 4A,B). Free Gem and SQ–Gem/EF NAs started to show tumor inhibitory effects 13 days after tumor cell inoculation. At day 27, free Gem treatment reduced the mean BLI signal value 1.70 times that in the control group. In mice treated with SQ–Gem/EF NAs, the tumor growth was significantly inhibited by 2.92 times (* *p* < 0.05) in comparison to the control group. The interweek tumor growth variation was also analyzed between days 20 and 27 (4th week) (Figure 4C). Results confirmed that SQ–Gem/EF NAs significantly restrained the tumor growth capacity in comparison to free Gem and Dex 5% treated groups in that interval.

At the endpoint of the experiment, micro-CT 3D images confirmed a marked ectopic bone formation (arrows) occurring to a similar degree in the Gem and non-treated groups (Figure 4E). Tumor-associated osteolytic lesions (asterisks) were especially visible around the knee region for all treatment groups. Nevertheless, less impact on the healthy bone was observed after treatment with the SQ–Gem/EF NAs, mostly near the femoral head area, and increased integrity of tibia and fibula bones could be observed. As expected, Ki-67-stained and hematoxylin and eosin-stained histological sections of the lungs revealed the presence of metastases. As seen in Figure 4D, six out of seven mice (85%) in the control group developed lung metastases. SQ–Gem/EF NAs and free Gem showed evidence of a major antimetastatic potential against OS: only two and three out of seven mice presented a single metastatic nodule in the SQ–Gem/EF NAs (28%) and free Gem (43%) treatment groups, respectively.

### 2.6. Safety Profile

The contribution of SQ–Gem/EF NAs treatment to toxicity in mice was also evaluated. Alanine aminotransferase (ALAT), aspartate aminotransferase (ASAT), creatinine and urea plasmatic levels were not noticeably higher or significantly different among treatments, indicating the absence of renal or hepatic disruption at the end of the experiment (Table 3). Only the creatinine level in the free Gem group was observed to be lower, compared to the non-treated or SQ–Gem/EF NAs group (0.063 versus 0.093 and 0.092 mg/100 mL, respectively). Compared to the control group, hematological analysis showed a markedly low blood cell count associated with the chemotherapeutic treatment, except for the platelet count. As observed in Table 3, the total white blood cell counts (WBC) were similar after free Gem and SQ–Gem/EF NAs treatments. However, the neutrophil total counts and their proportional WBC fraction were significantly more reduced in the free Gem group when compared to the control group. Importantly, although EF is a highly hemolytic compound, the red blood cell count (RBC) corresponding to the SQ–Gem/EF NAs treatment was found to be equal to that of free Gem treatment (7.93 versus 7.94 10^6^/µL). Moreover, only the hemoglobin concentrations (HGB) in the free Gem group were significantly lower in comparison with the control group.

## 3. Discussion

The prognosis for children and adolescents with high-grade bone sarcomas has remained unchanged for the past three decades [24]. In this study, we explored novel therapeutic opportunities offered by the incorporation of another antitumor agent (i.e., EF) into SQ–Gem NAs seeking to improve these patients’ quality of life. Thus, SQ–Gem NAs and SQ–Gem/EF NAs were successfully prepared and characterized in agreement with previous results [23]. Pharmacokinetic analysis showed that the equimolecular insertion of EF in the SQ–Gem nanoformulation triggered higher systemic clearance of SQ–Gem in mice in comparison to SQ–Gem NAs (Figure 2). Notwithstanding, the smoother hydrolysis of Gem from SQ–Gem/EF NAs indicated that the consequent minor AUC value of SQ–Gem was not associated with a burst release of Gem in the blood stream. This, together with the higher Vss value of SQ–Gem from SQ–Gem/EF NAs (Table 2), may suggest that SQ–Gem/EF NAs distribute towards peripheral tissues in a higher proportion than SQ–Gem NAs. In this regard, SQ–Gem from SQ–Gem NAs is known to bind to a large extent to blood stream components such as proteins and, singularly, lipoproteins [25]. Taking this into account—by using exclusively another antitumor agent (EF) as a scaffold for SQ–Gem—we were able to improve the tolerability of SQ–Gem NAs (Figure 3). From a physicochemical point of view, this is thought to be mainly related to the ability of EF to induce changes in the lipophilicity of the SQ–Gem NAs as well as in their molecular conformation. Hence, the in vivo fate of SQ–Gem/EF NAs could be influenced not only by their reduced size, but also because they may remain assembled for a longer time than SQ–Gem NAs. These findings support the notion of a chemotherapeutic strategy that is not commonly explored in the field of oncology. We must bear in mind that the reported maximum tolerated dose (MTD) of free Gem in mice is approximately 5 times higher than its equivalent dose of SQ–Gem NAs [19]. This is thought to be mainly associated with myelosuppression/hemotoxicity triggered by the long-term exposure of the total Gem pool (SQ–Gem plus the released Gem) in the blood stream, in agreement with preclinical toxicology data [19,26,27]. In the same way, the diminished AUC values of the total Gem pool, influenced by the incorporation of EF in the nanoformulation, correlated with lower toxicity of SQ–Gem/EF NAs compared to SQ–Gem NAs.

As previously stated, increasing the therapeutic dose may represent an opportunity for the treatment of aggressive solid tumors such as OS. We therefore selected a dosing schedule for SQ–Gem NAs 4 times higher than the tolerable dose previously described by Maksimenko et al. for the same mouse strain [28]. In our experimental conditions, the chemotherapeutic regimen by Maksimenko et al. may indeed run the risk of underdosing in our OS model, since Gem displayed anticancer activity in vitro at micromolar concentrations (Figure 1), when it is normally nanomolar in other OS cells [29]. A concurrent explanation could be related to the origin of these cell lines. Both cell lines were derived from genetically engineered mouse models designed to overexpress single oncogene c-Fos (P1.15 cells) and the heterozygous loss of the tumor suppressor p53 (p53^+/−^ OS cells). This single gene targeting could still preserve resistance mechanisms that are present in primary cells, narrowing the window for a therapeutic opportunity in these cells. Based on this initial in vitro screening, c-Fos overexpressing P1.15 osteoblasts were selected in order to reproduce high-grade pediatric OS in athymic nude mice. This proto-oncogene is related to the upregulation of fibroblast growth factor receptors, among other tumorigenic mechanisms, which involves an accelerated S-phase entry and a high propensity to migrate and invade other tissues [30,31].

In vitro, free Gem treatment was 35 and 6 times more active than free EF and SQ–Gem/EF NAs, respectively. Nonetheless, SQ–Gem/EF NAs were more effective at impeding the primary tumor progression in vivo (Figure 4). On the other hand, pulmonary metastases at diagnosis or especially during chemotherapy are associated with a dismal prognosis in pediatric OS [32]. In our study, SQ–Gem/EF NAs and free Gem treatments showed a similar ability to suppress the metastatic spread from the primary tumor to the lungs. An important fact that reinforces this approach is that EF is not an antimetabolite or an alkylating agent, which may be compatible with a more favorable quality of life in pediatric patients. However, free EF treatment was not included in the in vivo studies for ethical reasons due to its very high hemotoxicity [33,34]. This hinders the elucidation of any improvement in antitumor effect resulting from its action as antitumor agent beyond, for instance, a hypothetical biodistribution improvement of the NAs. In this regard, the reduced size of the SQ–Gem/EF NAs (i.e., 50 nm) may entail a high permeation effect around the leaky blood vessels present in the bone tumor area. Perhaps, it would be of interest to perform these experiments in a tumor model that is more sensitive to the action of EF. For instance, orally administered EF loaded into solid lipid nanoparticles proved to be very efficacious in an HOS (ATCC^®^ CRL-1543™) orthotopic tumor model [35], although the EF IC_50_ value in this cell line was 2.72 µM instead of 59 µM in the P1.15 cells.

Finally, SQ–Gem/EF NAs were safe, causing no apparent renal or hepatic damage (Table 3). Only the free Gem treatment group showed a significant slowdown in the weight gain of mice from day 21 (Figure 3B). Creatinine plasma levels were also lower in this group, suggesting a diminished metabolic activity, probably caused by the repeated administrations of Gem. Furthermore, hematological analysis revealed that the increased therapeutic efficacy of SQ–Gem/EF NAs was not associated with a worse dose/limiting myelosuppression in comparison with free Gem treatment. In fact, mice treated with SQ–Gem/EF NAs presented less neutropenia and anemia, which pose common restrictions on the use of Gem in the clinic [36]. This also confirmed the low hemolytic profile of SQ–Gem/EF NAs previously reported in vitro [23].

The vast majority of nanotechnology approaches for OS treatment to date rely on the anthracycline doxorubicin as a chemotherapeutic drug model [37]. Only two recent papers report the successful encapsulation of Gem in magnetite nanoparticles [38] and liposomes [39]. In the latter case, Caliskan et al. also proposed the combination of Gem with clofazimine, which is not a DNA disrupter per se like EF. However, to our knowledge this is the first time that a Gem-based nanoformulation (i.e., SQ–Gem/EF NAs) has proven anti-OS potential in vivo. Its safety profile and enhanced efficacy in delaying the primary tumor growth kinetics supports its implementation (for instance) in preoperative neoadjuvant chemotherapy protocols in order to effectively reduce the tumor burden [40].

## 4. Materials and Methods

### 4.1. Materials

Edelfosine (EF) was obtained from R. Berchtold (Biochemisches Labor, Bern, Switzerland). Gemcitabine (Gem) hydrochloride was purchased from Carbosynth, Ltd. (Compton, UK). Pure ethanol was purchased from Panreac Química (Barcelona, Spain). CellTiter 96^®^ aqueous one solution cell proliferation assay (MTS) and D-luciferin were purchased from Promega (Alcobendas, Spain). Heat inactivated fetal bovine serum, trypsin-EDTA, penicillin/streptomycin, alpha-minimum essential medium (alpha-MEM) and Phosphate Buffer Saline (PBS) were provided by Gibco^®^ (Invitrogen, Inc., Carlsbad, CA, USA). Difco™ Dextrose was obtained from BD Biosciences (Madrid, Spain).

### 4.2. Formulation and Characterization of Squalenoyl NAs

4-(N)-Tris-nor-squalenoyl–gemcitabine (SQ–Gem) was synthesized by linking the 1,1′,2-tris-nor-squalenic acid covalently to the amino group of the heterocyclic ring of Gem [17]. SQ–Gem/EF NAs and SQ–Gem NAs were formulated by the nanoprecipitation method as previously described [23]. Practically, a solution of SQ–Gem in ethanol alone or mixed with a solution of EF at equimolar concentrations was added dropwise into 2 mL of water (ratio ethanol/water 1:5) under stirring. Ethanol was evaporated in vacuum using a Rotavapor (Buchi R-210/215, Buchi Corp., New Castle, DE, US) to obtain an aqueous suspension of NAs ranging from 1.16 to 5.8 μmol mL^−1^. Mean particle size of the NAs, expressed in Z-average and PDI were determined at 25 °C by dynamic light scattering (Zetasizer Nano ZS Malvern; Malvern Instruments SA, Malvern, UK). Surface charge of the NAs was characterized by measuring the zeta potential with laser Doppler velocimetry (Zetasizer Nano ZS Malvern; Malvern Instruments SA, Malvern, UK).

### 4.3. Cell Culture and Cytotoxicity Assays

The P1.15 clonal OS cell line was cultured from a c-Fos/AP-1 murine transgenic OS [41]. p53^+/−^ OSs were obtained from a spontaneous OS developed in p53^+/−^ knock-out mouse. All cell lines were maintained in α-MEM cell medium, supplemented with 10% of inactivated fetal bovine serum and 1% penicillin/streptomycin in an incubator at 37 °C with a humidified atmosphere and 5% carbon dioxide.

For the in vitro cytotoxicity assays, p53^+/−^ OS and P1.15 cell lines were plated on 96-well plates at a density of 1000 cells per well. Twenty-four hours later, different treatments were added in triplicate for 72 h. After incubation with the different formulations, 100 μL of complete medium containing 15% v/v of MTS was added to each well. Absorbance was measured 2.5 h later in a microplate reader (Labsystem, Helsinki, Finland) at a test wavelength of 492 nm and 690 nm for the reference wavelength. The concentration of drug required to inhibit cell proliferation by 50% (IC_50_) was calculated using GraphPad Prism software (San Diego, CA, USA).

### 4.4. Pharmacokinetic Studies

Six-to-eight-week-old female athymic nude mice were obtained from Harlan Laboratory (Barcelona, Spain). Procedures involving animal handling and care were approved by the animal care and ethics committee (CEEA/031-18). A total of 24 mice were randomly assigned into two groups. To determine the pharmacokinetics, a single dose of either SQ–Gem NAs or SQ–Gem/EF NAs at 58 µmol/kg (Eq. to EF and SQ–Gem) in a dextrose 5% solution was injected via retro-orbital plexus (12 mice per group). This route is considered a suitable less stressful alternative to tail vein i.v. administration. Afterwards, 150 µL of blood was collected from the submandibular vein in EDTA surface-coated tubes (*n* = 3 at each time point) at various time points (5′, 15′, 30′, 1, 4, 8 and 24 h). Noncompartmental pharmacokinetic analysis was performed using the PKSolver add-in program for pharmacokinetics and pharmacodynamics data analysis [42].

Simultaneous quantification of Gem, SQ–Gem and EF in mouse plasma was performed using a UHPLC/MS/MS method as previously described [43]. The UHPLC system was composed of an Acquity UPLC^TM^ system (Waters Corp., Milford, MA, USA). Samples were chromatographically separated using an Acquity UPLC^TM^ BEH C_18_ column (50 mm × 2.1 mm, 1.7 m; Waters Corp.). Triple-quadrupole tandem mass spectrometric detection was performed on an Acquity^TM^ TQD mass spectrometer (Waters Corp.) with an electrospray ionization (ESI) interface. Calibration curves ranged from 0.05 to 125 μg/mL for SQ–Gem (R^2^ = 0.994) and from 1 to 100 μg/mL for EF (R^2^ = 0.995) and Gem (R^2^ = 0.996).

### 4.5. In Vivo Efficacy Studies

P1.15 cultured cells were harvested and diluted in PBS to a final concentration of 2 × 10^4^ cells per 5 μL. Intratibial injections were performed on six-to-eight-week-old female athymic nude mice (CEEA/031-18). Studies were performed at *n* = 7 per group, randomly allocated into experimental groups. Four days after tumor cell inoculation, animals received via retro-orbital plexus either free Gem or SQ–Gem NAs or SQ–Gem/EF NAs at 38 µmol/kg (Eq. to EF, SQ–Gem and Gem) or Dex 5% vehicle control twice per week, at intervals of 3 and 4 days during the experiment, for up to eight administrations.

P1.15 cells were genetically modified to express the luciferase gene, and the efficacy of the treatments was assessed by monitoring the primary tumor growth kinetics by BLI every week. Animals were anesthetized and inoculated with 50 μL of D-luciferin at 15 mg/mL. Images were taken for 1 min with a PhotonIMAGER™ imaging system (Biospace Lab, Nesles-la-Vallée, France) and analyzed using M3Vision software (Biospace Lab).

Histological analyses of the lungs were performed following fixation in a paraformaldehyde solution 4% in PBS. Paraffin sections were prepared for hematoxylin and eosin staining and Ki-67 immunohistochemistry. Aperio ImageScope software (Leica Biosystems, Nussloch, Germany) was used for histological visualization of lung metastases.

Micro-CT scan analyses of mice hindlimbs recovered in the necropsy were performed following fixation in a paraformaldehyde solution 4% in PBS. High resolution scanning for 3D imaging reconstruction was carried out using Computed Axial Tomography Quantum GX micro-CT imaging system, (PerkinElmer, Waltham, MA, USA) equipped with an X-ray source (voltage, 90 kVp, 8 W).

### 4.6. Clinical Chemistry and Hematology Analysis

Blood samples of mice were collected in EDTA surface-coated tubes prior to the necropsy. Quantification of liver enzymes, creatinine and urea in blood plasma was performed using a Roche Cobas c311analyzer. Blood counting analysis was performed using a Sysmex XT-1800i (Roche Diagnostics, Basel, Switzerland).

### 4.7. Statistical Analysis

Statistical analysis was performed using GraphPad Prism software (San Diego, CA, USA). Statistical comparison between different groups was performed using an ANOVA One-Way analysis of variance with Tukey post hoc correction. Data were reported as mean ± SEM/S.D., considering a 95% confidence interval at significance level * *p* < 0.05.

## 5. Conclusions

In this study, we investigated a new modality of squalenoyl nanocomposites for improving the therapeutic perspectives of high-grade solid tumors such as pediatric OS. It was shown that the equimolecular insertion of EF into SQ–Gem NAs drastically modified the exposure of SQ–Gem in the blood stream, which resulted in a better tolerability of the NAs after repeated i.v. administrations. Thus, the antitumor agent EF behaved as a modulator for SQ–Gem NAs, offering a novel therapeutic approach for the treatment of experimental aggressive pediatric solid tumors. Despite showing a modest anticancer profile in vitro in comparison to free Gem, SQ–Gem/EF NAs were able to efficiently decrease, in the absence of concomitant adverse effects, the kinetics of tumor growth and metastatic spread in P1.15 orthotopic OS mice. Further experiments are required to determine the optimal dosing schedule of SQ–Gem/EF NAs to better characterize their anticancer potential.

## Figures and Tables

**Table 1:** Colloidal characteristics of SQ–Gem and SQ–Gem/EF NAs.

NAs	Size (nm)	PDI	ζ (mV) ^1^	% Gem ^2^	% EF ^2^	% Total ^2^
SQ–Gem/EF	50 ± 4	0.12 ± 0.03	−12 ± 1	22.49	44.78	67.28
SQ–Gem	116 ± 10	0.09 ± 0.01	−17 ±1	40.7	0	40.7

^1^ Zeta potential. ^2^ % = MW drug/MW nanocomposites. The values are the mean ± SD (n ≥ 3).

**Table 2:** Comparison of pharmacokinetic parameters of SQ–Gem, Gem and EF after a single administration via retro-orbital plexus of SQ–Gem NAs or SQ–Gem/EF NAs (58 μmol/kg Eq. to EF and SQ–Gem). Values are means ± SD (*n* = 3).

Parameter	SQ–Gem NAs		SQ–Gem/EF NAs		
	SQ–Gem	Gem	SQ–Gem	Gem	EF
*t*1/2 (h)	2.12 ± 0.12	4.49 ± 0.47	2.37 ± 1.44	5 ± 0.6	30 ± 10
*C*max (μg/mL)	49 ± 13	3.3 ± 0.1	79 ± 10	3.65 ± 1.34	42 ± 6
Cl_obs (mL/kg/h)	462 ± 159	689 ± 47	948± 86	856 ± 18	86 ± 7
*V*ss_obs (mL/kg)	902 ± 280	4526 ± 165	1265 ± 694	7334 ± 1073	3534 ± 788
AUC 0-inf_obs (μg·h/mL)	88 ± 34	21 ± 1	39 ± 4	17 ± 0.38	346 ± 30

*t*1/2, terminal elimination half-life; *C*max, maximum observed plasma concentration; Cl_obs, systemic clearance; *V*ss, volume of distribution at steady-state; AUC 0-inf_obs, Area Under the curve from 0 extrapolated to infinite time. The values are the mean ± SD (*n* = 3).

**Table 3:** Evaluation of blood and plasma parameters in OS bearing mice on day 32 after eight retro-orbital plexus injections of either Dex 5%, free Gem or SQ–Gem/EF NAs at the dose of 38 μmol/kg (Eq. to EF, SQ–Gem and Gem).

Parameters	Units	Dex 5%	Free Gem	SQ–Gem/EF NAs
Plasma				
ALT	(UI/L)	20.3	24.2	17.8
AST	(UI/L)	177.8	129.4	141.5
Creatinine	(mg/100 mL)	0.093	0.063	0.092
Urea	(mg/100 mL)	42.1	45.6	37.2
Blood				
WBC	[10^3^/µL]	3.09	1.79 **	1.83 **
Neutrophils	[10^3^/µL]	1.56	0.54 ***	0.70 **
Neutrophils	(%)	50.59	31.60 **	39.54
RBC	[10^6^/µL]	8.82	7.93 *	7.94 *
HGB	[g/dL]	14.41	12.93 *	13.41
Platelets	[10^3^/µL]	711	753.5	710.85

ALT—alanine aminotransferase; AST—aspartate aminotransferase; WBC—white blood cell counts; RBC—red blood cell count; HGB—hemoglobin concentrations. Results expressed as means (*n* = 7), * *p* < 0.05, ** *p* < 0.01 and *** *p* < 0.001 versus Dex 5%.
